# Ghost guns and crime: a tale of two California cities

**DOI:** 10.1186/s40621-024-00500-w

**Published:** 2024-05-02

**Authors:** Alaina De Biasi, Anthony A. Braga, Brad Velasquez, Garen Wintemute

**Affiliations:** 1 Department of Criminology and Criminal Justice, Wayne State University, 656 W. Kirby St., Detroit, MI, 48202 USA; 2 Department of Criminology, University of Pennsylvania, 558 McNeil Building, 3718 Locust Walk, Philadelphia, PA, 19104-6286 USA; 3 Violence Prevention Research Program, Department of Emergency Medicine School of Medicine, University of California, 2315 Stockton Blvd, Sacramento, Davis CA, 95817 USA

## Abstract

**Background:**

Privately made firearms (PMFs) or “ghost guns” are homemade, unserialized, untraceable firearms that have been increasingly used in violent crime in the United States. Very little is known about the types of PMFs recovered by law enforcement agencies and the crimes associated with these recoveries. This lack of information limits effective violence prevention policies and practices. Comparative analysis of PMF recoveries in specific cities helps clarify whether local PMF patterns and characteristics vary or reflect more general trends. This research advances epidemiological understanding of emergent violent gun injury prevention challenges by identifying variations in recovered PMF types and use in violent, drug, and weapon-related offenses in Los Angeles and San Diego, California.

**Methods:**

Conjunctive analysis of case configurations (CACC) identifies patterns among observations (i.e., case configurations) and calculates their probability associated with a given outcome. CACC was used to identify the most common types of PMFs recovered by the Los Angeles (LAPD) and San Diego (SDPD) police departments. For each department and offense type, case configurations with above-average probabilities of offense involvement were determined. Comparisons across departments were made to identify similarities and differences in PMF characteristics and usage.

**Results:**

PMFs were more likely to be involved in violent and weapon-related offenses in Los Angles but more likely to be involved in drug-related offenses in San Diego. In both cities, the 9 mm Polymer 80 handgun was the dominant PMF. However, 9 mm handguns were most likely to be involved in weapon-related offenses in Los Angeles compared to 0.40 handguns in San Diego. Furthermore, large-caliber handguns tended to display above-average probabilities of involvement in violent and drug offenses in Los Angeles. Long guns were represented in case configurations with above-average probabilities of involvement in substantive crimes, including violence.

**Conclusions:**

Comparative analyses of PMF recovery patterns in Los Angeles and San Diego reveal meaningful contextual variations in PMF characteristics and suggest intentional firearm type selections by offenders. The results support increased regulation of PMFs and highlight the importance of efforts to identify and disrupt the illicit supply of large-caliber PMF handguns and PMF long guns.

## Background

Privately made firearms (PMFs) or “ghost guns” are homemade, unserialized, untraceable firearms that are produced from nearly finished precursor parts or 3D printed using software accessed for free online (Braga et al., 2022; Department of Justice [DOJ], 2022; Wintemute 2021a, 2021b). Individuals can purchase prepackaged kits with finishing and assembly instructions that include all the required parts to build a firearm. Alternatively, they may opt for partially complete kits or 80% kits, which necessitate the purchase of supplemental parts. With no record of their existence and minimal skill needed for their assembly, PMFs represent an attractive entry-point to illicit activity, especially for persons who are prohibited by federal or state laws from possessing a firearm.

Prohibited persons have traditionally acquired firearms through illegal diversions from lawful commerce, which include straw purchases, thefts, “off the book” transactions from federal firearms licensees (FFL), and, most commonly, informal transactions involving family members, friends, or street sources (Cook et al. 2015; Alper and Glaze 2016; Braga et al. 2021).^1^ These methods allow offenders to circumvent legal safeguards, such as a criminal background check, required for purchases from an FFL. Unlike PMFs, firearms acquired in such transactions are generally stamped with a serial number, a requirement set forth by the Gun Control Act of 1968. In combination with firearm purchase and transfer records preserved by FFLs, this feature allows law enforcement to follow transactions of serialized firearms from the point of their recovery to their most recent purchase from an FFL. Information gleaned from these investigations can help prevent and reduce firearm-related violent crime by identifying gun traffickers for apprehension and prosecution, and by contributing to epidemiological analyses of illegal gun market dynamics to inform strategies to shut down the flow of guns to prohibited persons and other high-risk individuals (Braga et al. 2002).

Until recently, PMFs have been unregulated at the federal level. Under these conditions, their use in illicit activities flourished. The number of suspected PMFs recovered in crimes in the United States (U.S.) and traced by the Bureau of Alcohol, Tobacco, Firearms and Explosives (ATF) increased by 1,083% (from 1,629 to 19,273) between 2017 and 2021, according to a report led by ATF (2022a).^2^ Among all states, the highest number of suspected PMFs were recovered in California, which accounted for 54.9% (20,875 of 37,980) of all suspected PMFs over the study period.^3^ Out of nearly 100 manufacturers, the company Polymer 80 contributed more than 88% (14,675 of 16,606) of suspected PMFs. In an earlier report, ATF (2022b) connected PMFs to 692 homicides or attempted homicides from 2016 to 2021.

Issued on April 11, 2022, the Final Rule (2021-R-05), Definition of “Frame or Receiver” and Identification of Firearms, sought to rectify the conditions that made PMFs attractive to offenders (DOJ, 2022). The ruling redefined a PMF to include “a firearm, including a frame or receiver, completed, assembled, or otherwise produced by a person other than a licensed manufacturer, and without a serial number placed by a licensed manufacturer at the time the firearm was produced” (p. 24,735). It also expanded the definition of a firearm to include firearm parts kits that are “designed to or may readily be completed, assembled, restored, or otherwise converted to expel a projectile by the action of an explosive” (p. 24,670). Under this new definition, the Final Rule requires the serialization of partially complete frames or receivers that are in an FFL’s inventory, as well as PMFs acquired by an FFL. FFLs are also required to perform criminal background checks on individuals who purchase a firearm parts kit and maintain purchase and transfer records of all PMFs.

Despite these newly placed legal safeguards, PMFs remain a formidable threat to public safety. The Final Rule immediately faced considerable legal challenges, with opponents proclaiming that ATF exceeded its regulatory authority (Thrush 2022). Ultimately, the ability of the Final Rule to stem the continued making of PMFs will be stymied without a federal court decision that affirms its legality. Compounding this threat, an unknown but undoubtedly very large number of unserialized firearm parts kits were sold prior to the Final Rule. Subsequent transfers of these illegal kits are very difficult to detect through the largely unregulated secondary firearm market. In addition, advances in 3D printing technology have made the production of PMFs more affordable and accessible to a larger audience (ATF, 2022a, 2022b).

The functionality of PMFs produced in this way has also markedly improved, due in part to the open-sourced nature of 3D printing software. This feature allows ongoing collaboration and design refinement to perfect the making of a wide array of firearm types and accessories, including assault weapons and Glock switches (or auto sears) that make firearms capable of fully automatic gunfire. Currently, 3D printing devices and software supporting the production of PMFs are unregulated.

The development of a knowledge base is a crucial first step toward informing injury prevention policies and practices. Early commentary provided by Wintemute (2021a, 2021b) alerted the public to the need for regulation, while also calling attention to our lack of knowledge on their impact on violent crime. ATF (2022a, 2022b) reports have helped initiate more formal discussions on the intensifying PMF problem by providing national-, state-, and select city-level estimates of suspected PMF recoveries and their characteristics.

What little is known about PMFs has been generated by two studies conducted in California. These studies focus on two Bay Area cities connected by a heavy-rail public transport system and a major highway. The first city-level study of PMFs was conducted by Braga et al. (2022), focusing on firearm recoveries in Oakland between 2017 and 2021. Over this period, the Oakland Police Department recovered 585 PMFs, predominantly 9 mm semiautomatic pistols. The proportion of PMFs among recovered crime guns dramatically increased from slightly more than 1% in 2017 to 24% in 2021. Further analysis revealed PMFs to be a weapon of choice for violent gun offenders. Approximately 17 miles south of Oakland, a second study observed a similar increase in the proportion of PMFs among recovered crime guns in Hayward, rising from less than 1% in 2015 to nearly 19% in 2021 (Jackson, Rippy & Chapin, 2023).

The intensifying public health and safety threat posed by the increasing prevalence of PMFs necessitates further comparative research into regional, state, and national trends. This study presents a comparative analysis of PMF recoveries in two very large southern California cities – Los Angeles and San Diego. The results can be used to clarify whether the Oakland and Hayward study findings reflect Bay Area regional patterns or more general PMF recovery trends. The State of California leads the nation in PMF recoveries (ATF, 2022a). As such, our results can also be used to frame epidemiological inquiries in other U.S. cities and states experiencing escalating PMF problems. Given existing laws and regulations, private persons can easily make customized firearms discreetly and economically throughout the U.S. As such, the characteristics of recovered PMFs may vary by crime type and across jurisdictions according to criminal preferences. Identifying existing nuances in PMF acquisition and use can better inform injury prevention policies and practices.

To this end, the current study extends prior research by exploring the patterns and characteristics of PMF recoveries by the Los Angeles (LAPD) and San Diego (SDPD) Police Departments and their association with violent, drug, and weapon-related offenses using the conjunctive analysis of case configurations (CACC) approach. An emergent method in criminology (e.g., Hart and Miethe 2015; Sytsma et al. 2021; Paez & Richmond, 2022), the CACC approach identifies patterns among observations, referred to as case configurations, and calculates their probability associated with a given outcome. The current study is motivated by two exploratory research questions to support its comparative review of PMF recoveries by LAPD and SDPD: (1) what are the most dominant types of PMFs?; and (2) what types of PMFs have an elevated likelihood of involvement in violent, drug, and weapon-related offenses?

## Methods

### Study site

Our city selections were informed by a supplemental ATF report on California that identified Los Angeles and San Diego as the top contributors of suspected PMFs from 2017 to 2021 (ATF, 2022c). Over this period, 2,602 suspected PMFs were recovered and traced in Los Angeles, and 936 were recovered and traced in San Diego, representing 12.4% (2,602 out of 20,875) and 4.5% (936 out of 20,875) of all suspected PMF recoveries in California, respectively.

Notable differences in crime, socio-economic, and geographic characteristics justified our comparative review of these two cities, with supporting data provided in Table 1. The average violent crime rate per 1,000 persons in Los Angeles over the study period was nearly double that of San Diego.^4^ Moreover, a comparison of relevant socio-economic characteristics obtained from the U.S. Census Bureau highlights significant differences between these cities.^5^ For instance, Los Angeles is more ethnically diverse than San Diego, with a larger population of foreign-born and smaller population of residents that identify as white alone. The city’s population is also less affluent, with a higher percentage of female-headed households and residents living below the poverty line, a lower percentage of residents with a high school degree or higher, and a lower median salary.^6^ In terms of geography, Los Angeles is much larger and more densely populated than San Diego. What is more, San Diego has a border with Mexico that spans approximately 60 miles, and its downtown is about 20 miles from the Mexican border. In comparison, the distance from downtown Los Angeles to the Mexican border is nearly seven times that length.

**Table 1:** Crime, socio-economic, and geographic characteristics

City Characteristics	Los Angeles	San Diego
Crime^a^		
Incidents of Part I Violent Crime, 2020	28,882	5,303
Incidents of Part I Violent Crime, 2021	30,078	5,770
Average Violent Crime Rate per 1,000 (2020–2021)	7.6	4.0
Percent Increase Violent Crime (from 2020 to 2021)	4.1%	8.8%
Population		
Population Estimates^b^	3,898,747	1,386,932
Foreign Born Persons^c^	36.3%	25.6%
Race		
White Alone^d^	34.9%	46.4%
Income and Poverty		
Persons in Poverty^e^	16.9%	11.8%
Median household income^f^ (In 2020 dollars)	$65,290	$83,454
Female-headed Households^d^	31.5%	27.4%
Education		
High School Graduate or Higher (25 years and over)^g^	78.3%	88.8%
Geography		
Population per Square Miles^b^	8,304.2	4,255.9
Land Area in Square Miles^b^	469.5	325.9
Estimated Distance from Southern Border to Downtown	137 Miles	20 Miles

^a^ Data was obtained from the FBI (2023) and the City of Los Angeles (2021)., ^b^ Data was obtained from the U.S. Census Bureau (2020 g). ^c^ Data was obtained from the U.S. Census Bureau (2020f). ^d^ Data was obtained from the Census Bureau (2020d). ^e^ Data was obtained from the Census and Bureau (2020e). ^f^ Data was obtained from the U.S. Census Bureau (2020c). ^g^ Data was obtained from the U.S. Census Bureau (2020b)

### Data

Our sample includes 3,147 PMFs that were recovered by LAPD and SDPD before the historic enactment of the Final Rule by ATF and were known to be involved in crime. Of these PMFs, 2,450 (77.9%) were recovered by LAPD over the period of January 1st, 2020, to October 31st, 2021, and the remaining 697 (22.1%) by SDPD over the period of January 1st, 2020, to December 31st, 2021.^7^

We obtained records containing information on the characteristics of recovered PMFs and the offenses associated with their recoveries from LAPD and SDPD. In these departments, it is standard protocol to record the identifiers (e.g., make, model, serial number), characteristics, and recovery details of *all* recovered firearms in a property collection database. While our study is limited by what is known about PMFs recovered by law enforcement, the comprehensive collection of data on PMFs by LAPD and SDPD constitutes a reasonable sample of PMFs used in crime (Cook and Braga 2001; National Research Council 2005).

Our study leverages these records to calculate the probability of involvement in violent (1 = violent offense; 0 = not violent offense), drug (1 = drug offense, 0 = not drug offense), and weapon-related offenses (1 = weapon-related offense, 0 = not weapon-related offense) across departments. Violent offenses involve the use or threatened use of force. Examples include assault, rape, robbery, and homicide. Drug offenses include activities related to the illicit production, sale, possession, and use of narcotics, as well as intoxication from alcohol and driving under the influence. Weapon-related offenses include violations of laws pertaining to firearms, such as the possession of a firearm by a known felon and carrying a concealed weapon without a license. The recovered PMFs in our sample may be associated with more than one of these offense types or some other offense type altogether.

To support our CACC analysis, we consider dichotomous measures in four domains that capture PMF characteristics: (1) firearm type (handgun or long gun, which includes shotguns and rifles), (2) manufacturer (80% Arms, James Madison Tactical, Palmetto Armory, Polymer 80, SS80, Strike Industries, or Unknown), (3) caliber (9 mm, 0.22, 0.223/5.56, 0.300, 0.308/7.62, 0.32, 0.357, 0.40, 0.45, 10 mm, 12 gauge, or Unknown) and (4) department (/recovery location) (LAPD or SDPD).^8^ We did not create an indicator for assault weapon given its high correlation with large-caliber long guns.

**Table 2:** Characteristics of PMF recoveries

Characteristic, *n* (%)	All (*N* = 3,147)	LAPD (*N* = 2,450)	SDPD (*N* = 697)
Firearm Type			
Handgun	2,906 (92.3)	2,308 (94.2)	598 (85.8)
Long Gun	241 (7.7)	142 (5.8)	99 (14.2)
Manufacturer			
Polymer 80	2,654 (84.3)	2,080 (84.9)	574 (82.4)
Unknown	456 (14.5)	335 (13.7)	121 (17.4)
SS80	27 (0.9)	25 (1.0)	2 (0.3)
Strike Industries	5 (0.2)	5 (0.2)	0 (0.0)
James Madison Tactical	3 (0.1)	3 (0.1)	0 (0.0)
Palmetto Armory	1 (0.0)	1 (0.0)	0 (0.0)
80% Arms	1 (0.0)	1 (0.0)	0 (0.0)
Caliber			
9MM	2,326 (73.9)	1,799 (73.4)	527 (75.6)
Unknown	371 (11.8)	332 (13.6)	39 (5.6)
0.4	216 (6.9)	172 (7.0)	44 (6.3)
0.223/5.56	162 (5.1)	92 (3.8)	70 (10.0)
0.45	36 (1.1)	26 (1.1)	10 (1.4)
0.22	17 (0.5)	15 (0.6)	2 (0.3)
0.308/7.62	8 (0.2)	4 (0.2)	3 (0.4)
10MM	5 (0.2)	5 (0.2)	0 (0.0)
0.357	3 (0.1)	3 (0.1)	0 (0.0)
0.3	2 (0.1)	1 (0.0)	0 (0.0)
12G	2 (0.1)	0 (0.0)	2 (0.3)
0.32	1 (0.0)	1 (0.0)	0 (0.0)
Violent Crime	467 (14.8)	358 (14.6)	109 (15.6)
Drug Crime	321 (10.2)	216 (8.8)	105 (15.1)
Weapon-related Crime	2,324 (73.8)	1,862 (76.0)	462 (66.3)
Other Crime	330 (10.5)	248 (10.1)	82 (10.8)

Note. Offense types are not mutually exclusive

### Analysis

We use the CACC approach to address our research questions. The CACC approach is informed by existing methods for multivariate analysis that explore relationships among dichotomous variables (for a review, see Miethe et al. 2008). As it relates to the current study, we consider four key domains of characteristics: firearm type, manufacturer, caliber, and department. Unlike multivariate contingency tables, the CACC approach considers the relative proportions of observations in the focal category of the outcome (i.e., Y[1 = present]), and provides a more concise representation of the related characteristics to facilitate comparisons.

The CACC approach begins with the development of a truth table. Captured as columns, the truth table includes all combinations of variable attributes, while their unique combinations are captured as rows. The truth table used in the current study includes a total of 336 potential configurations (2 [Firearm Type] x 7 [Manufacturer] x 12 [Caliber] x 2 [Department]). Once all possible configurations are identified, each observation is aggregated into its respective case configuration and two additional columns are generated. The first captures the total number of observations within a case configuration and the second the relative likelihood (or probability) of the outcome. For each case configuration, the CACC approach calculates the relative likelihood of the outcome by dividing the total number of observations for which the outcome is observed (i.e., Y[1 = present]) by the total number of observations for that case configuration. As applied to our study, the case configuration probabilities reveal the likelihood that a particular type of recovered PMF is involved in one of three offense types: violent, drug, or weapon-related.

Reflecting our consideration of three offense types, we create one table for each offense type that captures the total number of observations within a case configuration and their relative likelihoods. For ease of interpretation, we often refer to relative likelihoods as case configuration probabilities. To facilitate comparisons, we differentiate our findings by department (LAPD or SDPD). We then compare each case configuration probability to the average offense probability for the department. We calculate the average offense probability by dividing the total number of recovered PMFs involved in a particular offense by the total number of PMFs recovered by the department. Applying this calculation results in a total of 6 average offense probabilities (2 [Departments] x 3 [Offense Types]). Following Miethe et al. (2008), we focus our review on those case configurations probabilities that are greater than the average offense probability (hereon referred to as “above average”) and draw comparisons across departments.

Observations should cluster among a small number of configurations relative to the total number if strong contextual relationships exist among the case configurations (Hart et al. 2017). This phenomenon is referred to as situational clustering. For samples greater than 100, researchers have applied a minimum-frequency threshold of 10 or more observations per case configuration to identify situational clustering (e.g., Rennison et al. 2013; Sousa and Miethe 2010). More recently, formal measures of situational clustering have emerged, including a chi-square goodness-of-fit test and situational clustering index (SCI) (see Hart 2019). A statistically significant chi-square statistic and an SCI value close to 1 (on a 0–1 scale) are indicative of situational clustering. We evaluate situational clustering for each truth table and present findings on those case configurations with 10 or more observations.

## Results

### Characteristics of the sample

Table 2 provides descriptive statistics on all PMF recoveries and those made by each department. Mirroring prior research, handguns (92.3%; 2,906 of 3,147) were the most common firearm type and 9 mm the most common caliber (73.9%; 2,326 of 3,147). Polymer 80 accounted for 84.3% (2,654 of 3,147) of all PMFs, reflecting their dominance in the market. At 73.8% (2,324 of 3,147), the majority of PMFs were recovered from weapon-related offenses, followed by violent (14.8%; 467 of 3,147) and drug (10.2%; 321 of 3,147) offenses. These characteristics remained dominant when PMF characteristics were considered across departments. When compared across departments, three characteristics of PMFs are worth mentioning. First, the percentage of PMFs that are long guns is nearly three times larger for recoveries made in San Diego (14.2%) than for those made in Los Angeles (5.8%). Second, the percentage of PMFs involved in drug offenses in San Diego (15.1%) is nearly twice that of Los Angeles (8.8%). Third, a notably larger percentage of PMFs were involved in weapon-related offenses in Los Angeles (76.0%) than in San Diego (66.3%).

### Conjunctive analysis of case configurations

Table 3 presents the 16 case configurations that had 10 or more observations. A statistically significant chi-square goodness-of-fit test (*p* < 0.0001) and SCI value of 0.73 offer further evidence in support of situational clustering. Of these case configurations, 11 involved recoveries by LAPD; these 11 case configurations accounted for just 3.3% of 336 possible case configurations but 97.8% (2,397 of 2,450) of all LAPD recoveries. The remaining five case configurations involved recoveries by SDPD; these 5 case configurations accounted for 1.5% of 336 possible case configurations but 95.3% (664 of 697) of all SDPD recoveries. Our review of these 16 case configurations revealed the 9 mm Polymer 80 handgun as the weapon of choice, collectively accounting for 68.0% (2,141 of 3,147) of all observations.

**Table 3:** Case configurations with 10 or more recovered PMFs: LAPD and SDPD

Los Angeles Police Department (*N* = 2,450)					San Diego Police Department (*N* = 697)				
Case Configuration	Firearm Type	Manufacturer	Caliber	Total PMFs	Case Configuration	Firearm Type	Manufacturer	Caliber	Total PMFs
1	Handgun	Polymer 80	9MM	1,624	1	Handgun	Polymer 80	9MM	517
2	Handgun	Polymer 80	Unknown	246	2	Long Gun	Unknown	0.223/5.56	58
3	Handgun	Polymer 80	0.40	165	3	Handgun	Polymer 80	0.40	44
4	Handgun	Unknown	9MM	143	4	Long Gun	Unknown	Unknown	33
5	Long Gun	Unknown	0.223/5.56	75	5	Handgun	Unknown	0.223/5.56	12
6	Long Gun	Unknown	Unknown	50					
7	Handgun	Unknown	Unknown	27					
8	Handgun	SS80	9MM	21					
9	Handgun	Polymer 80	0.45	17					
10	Handgun	Polymer 80	0.22	15					
11	Handgun	Unknown	0.223/5.56	14					

Note. All PMFs were recovered by law enforcement

Tables 4, 5 and 6 display the results of our CACC findings for violent, drug, and weapon-related offenses, respectively. Case configurations are organized in descending order based on their relative likelihoods, with an asterisk indicating those that are greater than the average offense probability. These tables also include standard deviations, which indicate how much case configurations deviate from the average offense probability (± 1 SD). Our comparative review focuses on the top two case configurations for LAPD and SDPD across offense types.

**Table 4:** Case configuration (CC) probabilities: violent offenses

Los Angeles Police Department (*N* = 2,450) Average Offense Probability = 14.6%									San Diego Police Department (*N* = 697) Average Offense Probability = 15.6%						
CC	Firearm Type	Manufacturer	Caliber	PMFs involved in Violent Offenses	Total PMFs	Relative Likelihood		CC		Firearm Type	Manufacturer	Caliber	PMFs involved in Violent Offenses	Total PMFs	Relative Likelihood
1*	Handgun	Polymer 80	0.45	5	17	29.4%		1*		Handgun	Polymer 80	0.40	9	44	20.5%
2*	Long Gun	Unknown	0.223/5.56	19	75	25.3%		2*		Long Gun	Unknown	Unknown	6	33	18.2%
3*	Handgun	Unknown	Unknown	6	27	22.2%		3*		Handgun	Polymer 80	9MM	83	517	16.1%
4*	Handgun	Unknown	9MM	24	143	16.8%		4		Long Gun	Unknown	0.223/5.56	4	58	6.9%
5	Handgun	Polymer 80	9MM	237	1,624	14.6%		5		Handgun	Unknown	0.223/5.56	0	12	0.0%
6	Handgun	SS80	9MM	3	21	14.3%									
7	Handgun	Polymer 80	0.22	2	15	13.3%									
8	Handgun	Polymer 80	0.40	21	165	12.7%									
9	Long Gun	Unknown	Unknown	27	50	12.0%									
10	Handgun	Polymer 80	Unknown	5	246	11.0%									
11	Handgun	Unknown	0.223/5.56	1	14	7.1%									

Note. All PMFs were recovered by law enforcement. An asterisk identifies a case configuration with an above-average offense probability. For all case configurations, standard deviation = ± 7.2%

### Violent offenses

LAPD. Four case configurations had above-average probabilities of involvement in violent offenses; the average offense probability for all LAPD recoveries was 14.6% (358 of 2,450). These four case configurations captured 10.7% (262 of 2,450) of all PMFs recovered by LAPD, including 63 PMFs that were involved in violent offenses. Considering these PMFs, 71.3% (187 of 262) were handguns, 93.5% (245 of 262) were of an unknown manufacturer, and 54.6% (143 of 262) had a caliber of 9 mm. Furthermore, the top two case configurations involved 0.45 caliber Polymer 80 handguns (*n* = 5) and 0.223/5.56 long guns of an unknown manufacturer (*n* = 19) and had relative likelihoods of 29.4% and 25.3%, respectively. The relative likelihood for the top case configuration is more than twice as large as the average offense probability for the sample.

SDPD. Three case configurations had above-average probabilities of involvement in violent offenses; the average offense probability for all SDPD recoveries was 15.6% (109 of 697). These three configurations captured 85.2% (594 of 697) of all PMFs recovered by SDPD, including 98 PMFs that were involved in violent offenses. Considering these PMFs, 94.4% (561 of 594) were handguns manufactured by Polymer 80 and 87.0% (517 of 594) had a caliber of 9 mm. The top case configuration involved 0.40 caliber Polymer 80 handguns (*n* = 9), with a relative likelihood of 20.5%. This case configuration shares two attributes with the top case configuration for LAPD: handgun and Polymer 80. Furthermore, the case configuration that was the second most likely to be involved in a violent offense included long guns of an unknown caliber and manufacturer (*n* = 6), with a relative likelihood of 18.2%. This case configuration shares two attributes with the LAPD case configuration with the second highest relative likelihood: long gun and unknown manufacturer.

### Drug offenses

LAPD. Seven case configurations had above-average probabilities of involvement in drug offenses; the average offense probability for all LAPD recoveries was 8.8% (216 of 2,450), including 75 PMFs that were involved in drug offenses. These seven configurations captured 23.3% (572 of 2,450) of all PMFs recovered by LAPD. Considering these PMFs, 78.1% (447 of 572) involved handguns, 54.0% (309 of 572) were of an unknown manufacturer, and 56.4% (323 of 572) of an unknown caliber. The top case configuration involved 0.45 caliber Polymer 80 handguns (*n* = 4), with a relative likelihood of 23.5%. This type of PMF was also the most likely to be involved in violent offenses. At 18.0%, the case configuration with the second highest relative likelihood involved long guns of an unknown caliber and manufacturer (*n* = 9). This type of PMF was the second most likely to be involved in violent offense in San Diego.

**Table 5:** Case configuration (CC) probabilities: drug offenses

Los Angeles Police Department (*N* = 2,450) Average Offense Probability = 8.8%							San Diego Police Department (*N* = 697) Average Offense Probability = 15.1%							
CC	Firearm Type	Manufacturer	Caliber	PMFs involved in Drug Offenses	Total PMFs	Relative Likelihood	CC	Firearm Type	Manufacturer	Caliber	PMFs involved in Drug Offenses	Total PMFs	Relative Likelihood	
1*	Handgun	Polymer 80	0.45	4	17	23.5%	1*	Long Gun	Unknown	0.223/5.56	22	58		37.9%
2*	Long Gun	Unknown	Unknown	9	50	18.0%	2	Handgun	Polymer 80	9MM	73	517		14.1%
3*	Long Gun	Unknown	0.223/5.56	11	75	14.7%	3	Long Gun	Unknown	Unknown	1	33		3.0%
4*	Handgun	Unknown	0.223/5.56	2	14	14.3%	4	Handgun	Polymer 80	0.40	1	44		2.3%
5*	Handgun	Unknown	9MM	17	143	11.9%	5	Handgun	Unknown	0.223/5.56	0	12		0.0%
6*	Handgun	Polymer 80	Unknown	29	246	11.8%								
7*	Handgun	Unknown	Unknown	3	27	11.1%								
8	Handgun	Polymer 80	9MM	129	1,624	7.9%								
9	Handgun	Polymer 80	0.40	8	165	4.8%								
10	Handgun	SS80	9MM	0	21	0.0%								
11	Handgun	Polymer 80	0.22	0	15	0.0%								

Note. All PMFs were recovered by law enforcement. An asterisk identifies a case configuration with an above-average offense probability. For all case configurations, standard deviation = ± 10.0%

**Table 6:** Case configuration (CC) probabilities: weapon-related offenses

		Los Angeles Police Department (*N* = 2,450) Average Offense Probability = 76.0%						San Diego Police Department (*N* = 697) Average Offense Probability = 66.3%									
CC	Firearm Type		Manufacturer	Caliber	PMFs involved in Weapon-related Offenses	Total PMFs	Relative Likelihood	CC	Firearm Type	Manufacturer	Caliber	PMFs involved in Weapon-related Offenses		Total PMFs		Relative Likelihood	
1*	Handgun		SS80	9MM	18	21	85.7%	1*	Handgun	Polymer 80	0.40	31		44		70.5%	
2*	Handgun		Polymer 80	0.22	12	15	80.0%	2*	Handgun	Polymer 80	9MM	357		517		69.1%	
3*	Handgun		Unknown	0.223/5.56	11	14	78.6%	3*	Handgun	Unknown	0.223/5.56	8		12		66.7%	
4*	Handgun		Polymer 80	9MM	1,264	1,624	77.8%	4	Long Gun	Unknown	Unknown	19		33		57.6%	
5*	Handgun		Polymer 80	Unknown	189	246	76.8%	5	Long Gun	Unknown	0.223/5.56	28		58		48.3%	
6	Handgun		Polymer 80	0.40	125	165	75.8%										
7	Handgun		Unknown	9MM	101	143	70.6%										
8	Handgun		Unknown	Unknown	17	27	63.0%										
9	Long Gun		Unknown	0.223/5.56	47	75	62.7%										
10	Handgun		Polymer 80	0.45	10	17	58.8%										
11	Long Gun		Unknown	Unknown	28	50	56.0%										

Note. All PMFs were recovered by law enforcement. An asterisk identifies a case configuration with an above-average offense probability. For all case configurations, standard deviation = ± 10.3%

SDPD. Only one case configuration had an above-average probability of involvement in drug offenses; the average offense probability for all SDPD recoveries was 15.1% (105 of 697). This case configuration captured 8.3% (58 of 697) of all firearms recovered by SDPD, including 22 PMFs that were involved in drug offenses. Considering these firearms, 0.223/5.56 caliber long guns of an unknown manufacturer (*n* = 22) had the highest relative likelihood at 37.9%, which was more than twice as large as the average offense probability for the sample. This type of PMF was also the second most likely to be involved in violent offenses in Los Angeles.

### Weapon-related offenses

LAPD. Five case configurations had above-average probabilities of involvement in weapon-related offenses; the average offense probability for all LAPD recoveries was 76.0% (1,862 of 2,450). These five configurations captured 78.4% (1,920 of 2,450) of all LAPD recoveries, including 1,726 PMFs that were involved in weapon-related offenses. Considering these firearms, all were handguns, 98.2% (1,885 of 1,920) were manufactured by Polymer 80, and 85.7% (1,645 of 1,920) had a caliber of 9 mm. The top case configuration involved 9 mm SS80 handguns (*n* = 18), with a relative likelihood of 85.7%. At 80.0%, the case configuration with the second highest relative likelihood involved 0.22 caliber Polymer 80 handguns (*n* = 12).

SDPD. Three case configurations had above-average probabilities of involvement in weapon-related offenses; the average offense probability for all SDPD recoveries was 66.3% (462 of 697). These three configurations captured 82.2% (573 of 697) of all PMFs recovered by SDPD, including 396 PMFs that were involved in weapon-related offenses. Considering these PMFs, all involved handguns, 97.9% (561 of 573) were manufactured by Polymer 80, and 90.2% (517 of 573) had a caliber of 9 mm. The top case configuration involved 0.40 caliber Polymer 80 handguns (*n* = 31), with a relative likelihood of 70.5%. This type of PMF was also the most likely to be involved in violent offenses. At 69.1%, the case configuration with the second highest relative likelihood involved 9 mm Polymer 80 handguns (*n* = 357). The top two SDPD case configurations share two attributes with the LAPD case configuration with the second highest relative likelihood: handgun and Polymer 80. Like the top case configuration for LAPD, the case configuration with the second highest relative likelihood for SDPD also involved 9 mm handguns.

## Discussion

The scant available research suggests that PMFs are attractive to offenders because these unserialized firearms are easy to access and, after use in crime, cannot be subsequently traced by law enforcement agencies (e.g., Wintemute 2021a, 2021b; Braga et al., 2022). Our comparative and exploratory review of the characteristics of PMFs recovered by LAPD and SDPD, and their association with violent, drug, and weapon-related offenses, contributes to the development of a stronger knowledge base on PMFs in two substantive ways. First, the analyses develop a more refined account of the types of PMFs that may be more attractive for use in specific kinds of crime. Second, the use of PMFs in violent, drug, and weapons offenses seems to be powerfully shaped by regional contexts.

Our CACC analysis revealed patterns in the types of recovered PMFs most likely to be involved in violent, drug, and weapon-related offenses, suggesting intentional firearm type selections by gun offenders. For instance, case configurations involving 9 mm handguns were more likely to be involved in weapon-related offenses in Los Angeles, compared to 0.40 caliber handguns in San Diego. Furthermore, case configurations involving 0.40 and 0.45 caliber handguns tended to exhibit above-average probabilities of involvement in violent and drug offenses in Los Angeles. Despite their scarcity in our sample, long guns were also represented in case configurations with above-average probabilities of involvement in substantive crimes.

The identified patterns suggest that offenders acquire PMFs for specific purposes. A focused comparison of case configurations with above-average offense probabilities that involved 9 mm handguns to those that involved more powerful types of PMFs is particularly suggestive of this phenomenon. The 9 mm handgun is well-known for its concealability, reducing the chance of attracting undue attention from law enforcement officers. This feature contributes to its suitability for self-defense purposes (Sheley and Wright 1995; Hureau & Braga 2018). In comparison to bulkier, more powerful firearms, the 9 mm handgun may be more likely to be carried as a precautionary measure “in case” of unforeseen threats and subsequently deployed when such situations arise. The elevated likelihood of recovering a 9 mm handgun from incidents involving weapon-related offenses in Los Angeles aligns with this purpose and seems to reflect the daily risk of individuals being involved in violent interactions.

In comparison, we observed that large-caliber PMFs, specifically those designed for 0.40 and 0.45 caliber cartridge cases, as well as long guns, which include assault weapons, had above-average probabilities of involvement in violent and drug offenses. These types of PMFs are more lethal than the 9 mm handgun, underscoring the gravity of violent- and drug-related encounters, and the inherent and heightened risk of injury (Libby and Corzine 2007; Braga and Cook 2018). Put simply, individuals may be more likely to acquire PMFs with these characteristics when the likelihood of violence is high and there is a perceived need to neutralize threats by inflicting maximum damage. Furthermore, we identified case configurations with above-average probabilities of involvement in violent and drug offenses that shared one or more of these PMF characteristics. This observed overlap was present both within and across departments.

In addition to offender preferences, contextual differences across recovery locations further help explain the involvement of PMFs in violent, drug, and weapon-related offenses.

PMF recoveries by LAPD were more likely to be involved in violent and weapon-related offenses relative to PMF recoveries by SDPD.^9^ These differences could be attributed to higher levels of urban violence, promulgated by increased levels of community disadvantage, and by divergent policing styles. For example, during the study period, the average violent crime rate in Los Angeles was nearly twice as high as that in San Diego, which has a more affluent population. Furthermore, pretextual stops are a common tactic used by law enforcement to recover firearms (Rushin & Edwards, 2021). Data collected by the California Racial and Identity Profiling Advisory Board revealed that LAPD conducted nearly four times more traffic stops than SDPD over the study period (California Department of Justice, 2024). This very large disparity may in part explain the disproportionate recovery of PMFs by LAPD that were involved in weapon-related offenses.

As compared to Los Angeles, PMF recoveries that occurred in San Diego were more likely to be associated with drug offenses. As a Southwest border city, this finding may reflect the long standing active illegal drug and gun supply lines between San Diego and Mexico. Similarly, firearm trafficking between San Diego and Mexico may also influence the types of PMFs recovered by SDPD. To this point, an analysis of firearms seized by the Department of Homeland Security (DHS) identified rifles as the most common type of firearm trafficked between the U.S. and Mexico (U.S. Government Accountability Office, 2021). In comparison, ATF analyses of firearms trace data found that rifles were the second most frequently recovered firearms in Mexico that originated from FFLs in the U.S (ATF, 2021).^10^ Goodman and Marizco (2010) further documented the desirability of rifles by Mexican drug trafficking organizations in an analysis of firearm trafficking cases with a San Diego nexus. The disproportionate recovery of long guns in San Diego and their connection to drug offenses align with this established pattern.

### Limitations

The current study is not without limitations. An inherent limitation of research on recovered crime guns is sample selection bias. Law enforcement only recovers a small fraction of guns involved in criminal activities, a factor influenced by the extent of proactive policing activities (Cook and Braga 2001). Notwithstanding this issue, LAPD and SDPD collect comprehensive data on all recovered crime guns. The National Research Council (2005) suggests that comprehensive crime gun data can be used in valid descriptive and policy evaluation research when the appropriate care is taken to recognize data limitations. Future studies should carefully consider the limits of police data collection practices when conducting research on recovered crime guns. Furthermore, our comparison of two California cities limits our ability to generalize our findings to other contexts. Nevertheless, the observed variations between cities suggest that exploring the types of PMFs used in crime in other contexts is a worthwhile endeavor. For example, the restrictiveness of state firearm laws and the movement of PMFs within and across states and internationally are factors that may drastically impact patterns in PMF recoveries by law enforcement agencies. These influences could be revealed through comparative analyses. Our CACC approach also precludes us from making statistical inferences. Despite these limitations, these data are sufficient for exploratory purposes. Future research can expand our work by using the patterns revealed from case configurations of PMF recoveries to inform inferential assessments across different jurisdictional contexts. Future research should compare the characteristics of PMFs and non-PMFs recovered by law enforcement to reveal similarities and differences in offense involvement by manufacturing pedigree.

## Conclusions

Our study serves as a valuable guide for future epidemiological analyses of PMFs, providing insights that can inform supply-side violence reduction strategies. We found distinctive patterns in the types of PMFs involved in violent, drug, and weapon-related offenses in Los Angeles and San Diego. These patterns suggest that the characteristics of PMFs not only reflect offender perceptions of the likelihood and severity of criminal encounters but are also influenced by the context in which these encounters occur. These findings support the regulation of PMFs through ATF’s Final Rule, which could possibly have a profound impact on the recovery of PMFs through serialization of firearm parts and assembly kits such as 9 mm Polymer 80 handguns—the most frequently recovered PMF by both LAPD and SDPD. Beyond regulation, these results support enhanced law enforcement efforts to identify and disrupt local sources of large-caliber PMFs and long guns, given their lethality and heightened likelihood of involvement in substantive crimes. Such attention may be especially impactful in Southwest border cities like San Diego, which are disproportionately affected by firearm and drug trafficking between the U.S. and Mexico.

## Data Availability

The datasets analyzed on PMF recoveries are not publicly available. The authors are unable to share these specific datasets due to constraints outlined in their data sharing agreements. The datasets analyzed on violent crime are available in the FBI data repository, https://cde.ucr.cjis.gov/LATEST/webapp/#/pages/home. The datasets analyzed on socioeconomic characteristics are available from the U.S. Census Bureau, https://www.census.gov/.

## Footnotes

1 A straw purchase is defined as “…the illegal purchase of a firearm by one person for another” (ATF, 2018).

2 ATF defines a suspected PMF as an “unserialized firearm that has been recovered in a criminal investigation, submitted to ATF for tracing, and determined to likely have been privately made” (ATF, 2022a, p. 54).

3 California’s ranking as the top state in suspected PMF recoveries is unlikely due to better recovery efforts. Instead, it is more likely a result of the tightly regulated nature of its licit firearm market.

4 We collected data on Part I violent crimes that occurred in 2020 in Los Angeles and 2020 and 2021 in San Diego from the Federal Bureau of Investigation’s (FBI) (2023) crime data explorer, an open-sourced data portal. Data on Part I violent crimes that occurred in 2021 in Los Angeles were unavailable. Consequently, we obtained an estimate provided by the City of Los Angeles (2021). To calculate violent crime rates, we used population estimates for Los Angeles and San Diego obtained from the U.S. Census Bureau (2020f).

5 The sociodemographic and geographic characteristics discussed here were obtained from the U.S. Census Bureau (2020a-f).

6 Los Angeles and San Diego fared similarly in terms of the percentage of male residents, residents under the age of 18, and residents between the ages of 18 and 24 (see U.S. Census Bureau, 2020a).

7 At the time of the data request, LAPD was only able to provide records of recovered PMFs through October 31st, 2021. Consequently, our sample constitutes a convenience sample.

8 Unknown manufacturer and caliber information should not be interpreted as missing data. This categorization is common in trace reports of PMFs due to their lack of required markings and law enforcement’s unfamiliarity with them (ATF, 2022a).

9 While the likelihood that PMF recoveries by LAPD involved violent offenses is only slightly higher than SDPD, the difference between the offense probabilities of the above-average case configurations and average violent offense risk is much larger for LAPD than SDPD.

10 HSI’s analysis of firearms trafficked from the U.S. to Mexico focused exclusively on firearms seized within the U.S. by DHS that were intended for Mexico. These data were not considered by ATF in a comparable analysis of firearms trafficked from the U.S. to Mexico (U.S. Government Accountability Office, 2021).

## Abbreviations

ATF: Bureau of Alcohol, Tobacco, Firearms, and Explosives
CACC: Conjunctive analysis of case configurations
DHS: Department of Homeland Security
FBI: Federal Bureau of Investigation
FFL: Federal Firearms Licensees
LAPD: Los Angles Police Department
PMF: Privately made firearm
SCI: Situational clustering index
SDPD: San Diego Police Department
U.S: United States
